# Correlation of the basic reproduction number (*R*_0_) and eco-environmental variables in Colombian municipalities with chikungunya outbreaks during 2014-2016

**DOI:** 10.1371/journal.pntd.0007878

**Published:** 2019-11-07

**Authors:** Víctor Hugo Peña-García, Rebecca C. Christofferson

**Affiliations:** 1 Programa de Estudio de Control de Enfermedades Tropicales (PECET), Facultad de Medicina, Universidad de Antioquia, Medellín, Colombia; 2 Department of Pathobiological Sciences, School of Veterinary Medicine, Louisiana State University, Baton Rouge, Los Angeles, United States of America; Yale School of Public Health, UNITED STATES

## Abstract

Chikungunya virus (CHIKV) emerged in Colombia in 2014 into a population presumed fully susceptible. This resulted in a quick and intense spread across Colombia, resulting in an epidemic that affected an estimated 450,000 people. The reported Colombian cases accounted for over 49% of all the cases reported to the PAHO. Eco-environmental factors are known to be associated with the spread of arboviruses such as CHIKV, and likely contribute to the differences in transmission profiles that were observed across several municipalities. To determine the association of eco-environmental factors and CHIKV, the basic reproduction number (*R*_0_) in 85 municipalities, which accounted for 65.6% of reported CHIKV cases in Colombia, was estimated. Estimates of *R*_0_ ranged from 1 to 9, where over 76% of municipalities had *R*_0_ values between 1 and 2. When we looked at the distribution of *R*_0_, the cumulative proportions were 20% with *R*_0_>2, 14% with *R*_0_>3, and 9% with *R*_0_>4. Next, we determined that there were different patterns of correlation between environmental and/or ecological variables and *R*_0_ when we considered different *R*_0_ lower-thresholds. Broadly, we found that temperature-related variables are significantly and positively correlated to *R*_0_ regardless of the lower threshold, while other variables like duration of outbreak and size of the urban area are inversely related to *R*_0_. Specifically, we conclude that high values of temperature-related variables where *R*_0_ > 1 will result in a fast growth of cases in a shorter time period (with faster cessation of outbreak transmission) but will result overall in a fewer total cases compared to outbreak areas (*R*_0_ > 1, but classified as lower). Thus, in the absence of vector control, a less explosive outbreak may be more advantageous for the virus in terms of transmission.

## Author summary

Chikungunya virus emerged in Colombia in 2014 into a presumed fully susceptible population and rapidly spread in the country. Numerous municipalities were differently affected by this virus across the country. The main purpose of this work was understanding why those differences were produced and, in turn, what are the variables addressing such differences. For this purpose, we estimated for 85 municipalities the basic reproduction number (*R*_0_), a crucial parameter to understand epidemics that is expressed as the number of secondary cases produced by a primary case. Such parameter was correlated with numerous variables resulting evident a crucial role of temperature in the increase of *R*_0_. Interestingly, other variables like size of the urban area and cases showed to be negatively correlated with *R*_0_. Results shows that high temperatures produce high *R*_0_, but those municipalities that showed high *R*_0_ showed an explosive epidemic with faster increase of cases that ceased equally fast, so the duration of epidemic is short producing small amount of cases. In this way, more cases are expected with municipalities with lower values of *R*_0_, which is suitably explained by the tortoise-hare model, where the less explosive outbreak results to be more advantageous for the virus.

## Introduction

Chikungunya (CHIKV) is an arbovirus that causes a febrile illness, and belongs to the family Togaviridae and genus *Alphavirus*. CHIKV is transmitted by vectors belonging to genus *Aedes*, primarily *Ae*. *aegypti* and *Ae*. *albopictus* [1]. CHIKV had been limited to Asia and Africa until 2007 when the virus reached Europe, causing sporadic outbreaks, and finally the Americas in 2013, where it quickly caused over 1 million cases [2]. CHIKV emerged in Colombia in 2014, where the population was generally naïve. The subsequent Colombian epidemic of CHIKV accounts for approximately 49.15% of cases in Latin America as reported by the Pan American Health Organization (PAHO) [3].

One of the metrics used to describe the transmission dynamics of infectious diseases is the basic reproduction number (*R*_0_), which is defined as the average number of secondary cases generated by a primary case introduced into a fully susceptible population [4, 5]. It has been suggested for vector-borne pathogens that *R*_0_ is the expected number of people that will be infected by one person initially infected by a vector [6, 7]. It is theorized that an epidemic occurs when *R*_0_ is greater than or equal to 1, although an outbreak is possible even with *R*_0_ lower than 1 [7].

The generation time, which is the time between the infection of a primary case and one of its secondary cases, is crucial to estimate of *R*_0_ but this value is often unknown [8]. Alternatively, a parameter called the serial interval has been described and is defined as the elapsed time between the onset of the symptoms of the primary and the secondary cases. This interval is more consistently observable in settings where clinical manifestation leads to case detection, but the serial interval is not readily extracted from epidemiological data [8]. Alternatively, some studies of arboviruses have assumed that the serial interval is the sum of estimates of the extrinsic incubation period of a virus (within the mosquito) and the intrinsic incubation periods of a virus (within the vertebrate host) [9, 10]. This is appropriate, as it takes into account the properties of virus transmission both within the vector as well as the vertebrate and is the definition for serial interval we use herein. However, it is important to acknowledge that while the intrinsic incubation period for CHIKV has been estimated to be approximately 3 days, there are several estimates of the extrinsic incubation period, which depend on other extrinsic factors, most notably temperature, leading to a range of possible serial intervals [11–15].

While *R*_0_ describes the epidemic potential of a pathogen, it can also be used to investigate modulation of this potential by external factors. For example, temperature is a known effector of mosquito-borne diseases, particularly as it relates to the vector-virus interaction [16–19]. Since arboviral transmission is affected by environmental and ecological variables, we wanted to determine whether any pattern could be discerned from administrative level incidence data in Colombia. Accordingly, we estimated *R*_0_ of CHIKV from the Colombian National Institute of Health (INS) and investigated the correlation with many potentially influential eco-environmental variables to determine if and how they influence the CHIKV spread and incidence in an outbreak scenario. Further, given the differences in transmission dynamics across the municipalities, we investigated the discrepancy between the magnitude of *R*_0_ and the number of cases.

## Methods

### Estimation of *R*_0_ and variables of interest

*R*_0_ was estimated using the White-Pagano methodology, which was shown to be consistent with other methodologies [9, 20]. However, there are advantages to the White-Pagano method when estimates must be made on administrative incidence data. An important advantage of White-Pagano method is that serial interval can be directly approximated from epidemiological data. Given that the White-Pagano methodology allows for the estimation of *R*_0_ without a priori observation of the exact serial interval, it is suitable for the estimation of *R*_0_ for different municipalities with different arboviruses transmission conditions, such as temperature profiles, where the serial interval likely varies. However, the WP method does require an estimated range of possible serial intervals. Herein we assumed a possible serial interval of 1 or 2 weeks so that the unit of time matched the incidence data (as we do not know the distribution of cases in days within each epidemiological week), which allows for some range in estimation of CHIKV serial interval that could be related to variability in EIP [21–24]. The likelihood-based method used for the estimation of the *R*_0_ and the serial interval (time between the onset of symptoms of first case and the onset of symptoms of the secondary case [8]) is as follows:

$$L(R_{0},p)=\prod_{t=1}^{T}\frac{e^{-\mu_{t}}{\mu_{t}}^{N_{t}}}{N_{t}!}$$

where $\mu_{t}=R_{0}\sum_{j=1}^{min(k,t)}N_{t}-jP_{j}$, *N*_*t*_ is the incidence data; *P*_*j*_ is the serial interval with *j* = 1, …, *k*; and *t* is the time of epidemiological data. Using this methodology, it is possible to estimate both *R*_0_ and *P*_*j*_ by using maximum likelihood techniques [20]. Thus, the White-Pagano methodology allows for estimation of the *R*_0_ even when the serial interval is not known *a priori*, which makes this methodology suitable for the estimation of *R*_0_ for arboviruses in different municipalities, i.e. with different temperatures which can modify the length of the extrinsic incubation period within the mosquito vector. However, this methodology is sensitive to the total number of cases in the system. Thus, we selected the Colombian municipalities with the highest number of cases in order to confidently apply this derivation of *R*_0_ to comparisons across environmental variables of the municipalities [20].

With the range of serial intervals used to initiate the model of 1–2 weeks, we assumed a binomial distribution for the serial interval for all municipalities [20]. The analysis determined the probability of having a 1- or 2-week serial interval for each municipality and assigned the serial interval with the highest probability (S1 Fig). Additionally, given the 2-week maximum serial interval, case counts were included in the determination of the epidemic curve per municipality if new cases occurred within two weeks of the previous case. If a case occurs outside of this two-week window, it was considered sporadic or possibly the results of a new introduction and not included. Having shaped the epidemic curve for each local epidemic, the estimation of *R*_0_ included cases starting from the week of the first case until the local peak of the epidemic. The outbreak decline phase of the epidemic is not taken into account for the *R*_0_ estimation using this methodology [20].

### Epidemiological and demographic data

Epidemiological data was obtained from Colombian INS which publishes through its web page (www.ins.gov.co) the reported cases collected by the national system of epidemiological surveillance (*SIVIGILA*). Data for CHIKV cases was taken for the years 2014 to 2016. Data collected through SIVIGILA is reported on a weekly basis, i.e. weekly number of chikungunya cases per city reporting. Population projections developed by the Colombian National administrative department of Statistics (*DANE* according to its name in Spanish) were used to estimate population size per city and subsequently incidence. Incidence was estimated per 100,000 individuals per each city. Urban area size (termed “urbanicity”) and map resources were obtained from the National Geostatistical Framework (*MGN*, according to its name in Spanish) of DANE.

### Environmental data

Temperature data of each city were extracted from Worldclim climatic layers [25]. We used data of minimum temperature, maximum temperature and average temperature by using the coordinates taken from the center of the urban area of each municipality that were obtained from the software Google Earth Pro version 7.3.2.5491 (Google LLC, Mountain view, California, USA). The difference of temperature (maximum temperature minus minimum temperature) was estimated from that data.

For each municipality, we calculated the number of cumulative cases until epidemic peak (“Peak cCases”), the time to onset of detected outbreak in the municipality (the first week with reported cases in the municipality relative to the first week with reported cases in Colombia, “outbreak onset”), and duration of the outbreak (in weeks).

### Statistical analysis

All analyses were performed in *R* version 3.5.1. After *R*_0_ was estimated as above, we correlated the values of environmental and demographic variables with the *R*_0_ estimate using Spearman’s correlation across all municipalities. Correlations with *p*<0.05 were considered significant. Following this, we wanted to determine if there was a differential pattern of corollaries dependent on the intensity of transmission. As *R*_0_ is a threshold parameter (lower bound of 1 to indicate outbreak likelihood), we did this by altering the (lower) threshold to indicate differential intensity of transmission. Thus, we conducted the same Spearman’s correlation analysis for the following subsets of municipalities: *R*_0_ > 2, *R*_0_ > 2.5, and *R*_0_ > 3. These were chosen based on the distribution of *R*_0_ (see Results below).

We then wanted to determine if the variable values (such as temperature) were differentially associated depending on the *R*_0_ value. For this, we divided municipalities into three pairwise comparisons: 1 < *R*_0_ ≤ 2 versus 2 < *R*_0_; 1 < *R*_0_ < 2.5 versus 2.5 < *R*_0_; and 1 < *R*_0_ < 3 versus 3 < *R*_0_. Comparisons were done using Wilcoxon rank sum test.

Maps were created in ArcGIS 10.4.1 from publicly available dataset described above and from our calculation of *R*_0_ from those datasets.

## Results

### Estimation of *R*_0_ from CHIKV incidence data

**Fig 1:**
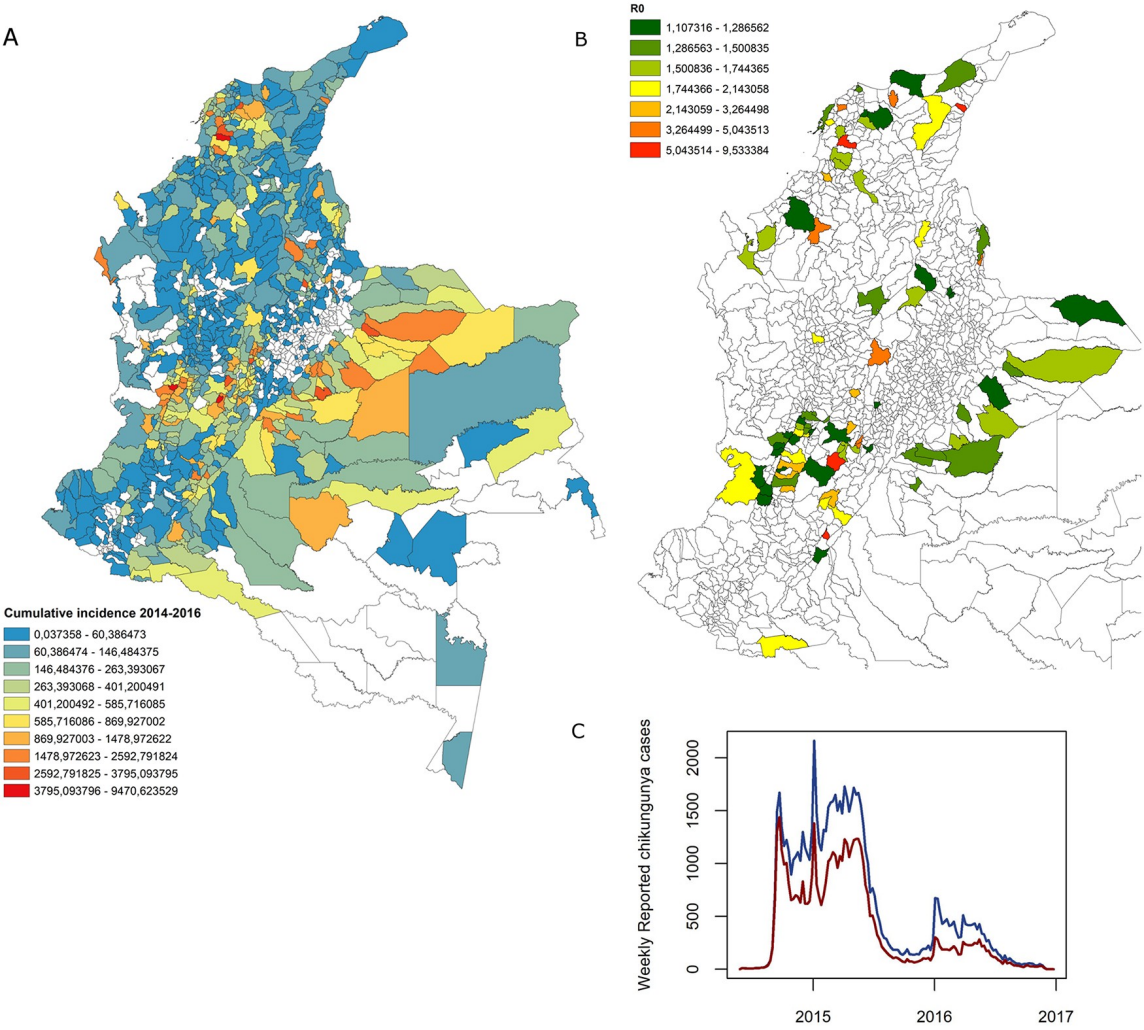
General behavior of the chikungunya epidemic that occurred in Colombia from 2014 to 2016. (A) Cumulative incidence discriminated by municipalities in Colombia according to cases reported through SIVIGILA by INS, (B) *R*_0_ estimated for 85 municipalities with epidemic CHIKV in Colombia and their geographical locations, (C) Behavior in time of the total reported cases in Colombia (blue line) and the reported cases of the 85 municipalities chosen to estimate *R*_0_ (red line). All maps were generated using ArcGIS 10.4.1 for this publication by the authors and has not been published elsewhere.

From 846 municipalities that reported chikungunya cases between 2014 and 2016 we selected 85 that had a recognizable epidemic peak and reported more than 150 cases. The chosen municipalities comprised approximately 65.6% of Colombian reported cases (Fig 1). From those municipalities, the range of cases reported over the two years of peak CHIKV incidence in Colombia was 151 (city of Soledad) to 4179 cases (city of Cali). The city of Turbaco reported the highest number of cases in a single week, which was 589 cases.

The *R*_0_ in the 85 municipalities ranked from 1.11 in Floridablanca (Department of Santander) to 9.53 in the municipality of El Molino (La Guajira department). Most of the municipalities were estimated to have a one-week serial interval rather than a two-week serial interval (S1 Fig). The distribution of *R*_0_ values estimated are given in S2 Fig. Estimates of *R*_0_ ranged from 1 to 9, where over 76% of municipalities had *R*_0_ values between 1 and 2. When we looked at the distribution of *R*_0_, the cumulative proportions were 20% with *R*_0_>2, 14% with *R*_0_>3, and 9% with *R*_0_>4.

**Fig 2:**
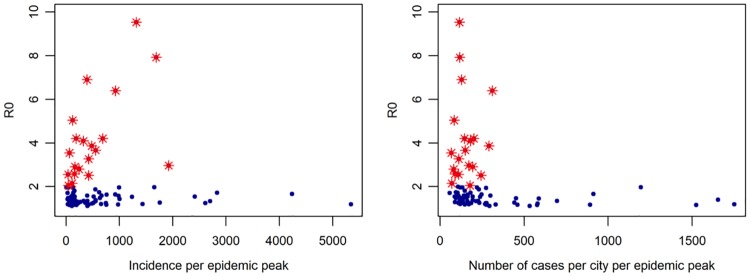
Relationship of *R*_0_ and cumulative CHIKV incidence of 85 municipalities during their respective outbreaks between 2014 and 2016. *R*_0_ higher than 2 are highlighted in red while blue indicates those municipalities with 1 < *R*_0_ < 2.

When we visualized the estimated *R*_0_ versus CHIKV incidence in each municipality, we found the relationship to be relatively dichotomous with an apparent break at approximately *R*_0_ > 2 (Fig 2). While the relationship between incidence and *R*_0_ is obvious, the stark separation shown by this relationship was surprising and further lead us to investigate alternate lower bounds of 2.5 and 3 (see below).

### Correlation of *R*_0_ and environmental/ecological variables when lower bound or *R*_0_ is varied

Table 1 outlines the results of the correlation analysis of 13 variables. When all municipalities were considered, the number of cases, mean, max, and min temperature, as well as the duration of the epidemic and size of urban area were all significantly correlated to *R*_0_. Similar to what is seen in Fig 2, there was a surprising negative correlation between *R*_0_ and the duration and number of cases. Temperature was positively associated with *R*_0_ values.

**Table 1:** Spearman correlation coefficients (ρ) between *R*_0_ and different variables when the lower threshold of *R*_0_ is 2, 2.5, or 3.

Variable	*R*_0_>1	*R*_0_>2	*R*_0_>2.5	*R*_0_>3
Peak cCases	***-0*.*3758***	0.144	0.110	0.490
Mean temperature	***0*.*3037***	0.433	0.344	-0.580
Max temperature	***0*.*3667***	***0*.*520***	0.387	-0.322
Min temperature	***0*.*2664***	0.245	0.243	***-0*.*636***
Temperature range	0.1187	0.283	0.034	0.322
Total population	-0.1868	***-0*.*489***	-0.406	-0.329
Urban population	-0.1698	***-0*.*469***	-0.389	-0.308
Incidence	0.0167	***0*.*571***	0.439	0.476
Duration until peak	-0.202	-0.340	-0.140	0.064
Total duration	0.0457	-0.164	0.146	-0.050
Outbreak OnsetWeek after first	***-0*.*227***	-0.438	-0.278	-0.046
Urban area	0.0123	-0.385	-0.247	-0.161
Population density	***-0*.*3758***	-0.409	-0.418	-0.455

Italic bold indicates statistically significant at 95% confidence level.

We determined that the magnitude and direction of the correlation coefficients of some variables changed according to the lower bound of *R*_0_ (Table 2). When the lower bound of *R*_0_ was greater than 2, the maximum temperature, total population, urban population, and incidence were significant. Number of cases and duration of outbreak was not significantly correlated with the value of *R*_0_, indicating that the relationship between the shape and size of the epidemic and *R*_0_ is driven mostly by those outbreaks where 1≤*R*_0_≤2. As the lower bound was further raised to 2.5 and 3, correlation with these variables was not statistically significant. The only exception was a statistically significant and negative correlation between *R*_0_ and the minimum temperature when the lower bound was 3, indicating that the biological trend of higher temperatures leading to more transmission is still apparent, but not captured with maximum temperature solely.

**Table 2:** *P*-value of Wilcoxon sum rank test with different variables between municipalities separated according to groups where *R*_0_ > of 2, 2.5 or 3 compared to groups where *R*_0_ ≤ 2, 2.5, or 3.

Variable	*Comparison of R0> 1 with altered lower threshold of*		
	*R*_0_>2	*R*_0_>2.5	*R*_0_>3
Number of cases	***0*.*0205***	***0*.*0448***	0.1612
Mean temperature	0.106	0.0786	***0*.*006***
Maximum temperature	***0*.*0291***	***0*.*0144***	***0*.*0008***
Minimum temperature	0.1953	0.216	***0*.*023***
Difference of temperature	0.1264	***0*.*0222***	0.1315
Population size	0.2274	0.1126	0.0872
People in urban center	0.19	0.0965	0.0591
Incidence during epidemic	0.9958	0.5505	0.3218
Week after first case	0.5723	0.246	0.1088
Duration	***6*.*552e-08***	***7*.*312e-08***	***2*.*21e-05***
Size of urban area	***0*.*0449***	***0*.*0204***	***0*.*0234***
Density population	0.3472	0.5025	0.9745

Significant differences are highlighted in italic bold.

### Association of *R*_0_ when lower bound is modified with eco-environmental variables

The Wilcoxon rank sum test showed that there were differences in several variables when compared to groups of “low” versus “high” based on the lower bound of the *R*_0_ estimates (Table 2). We found consistent differences in the distribution of number of cases when all municipalities were considered (lower threshold *R*_0_ = 1) versus the modified lower thresholds of 2 or 2.5. Mean, minimum, and difference in temperature was not consistently different across all comparisons. Mean and minimum temperature were only significant between the comparison of *R*_0_>1 versus *R*_0_>3; difference in temperature was only significant in the comparisons of *R*_0_>1 versus *R*_0_>2.5. Maximum temperature was different among all comparisons, indicating that there is a consistent relationship between this variable and increasing *R*_0_ estimates.

**Fig 3:**
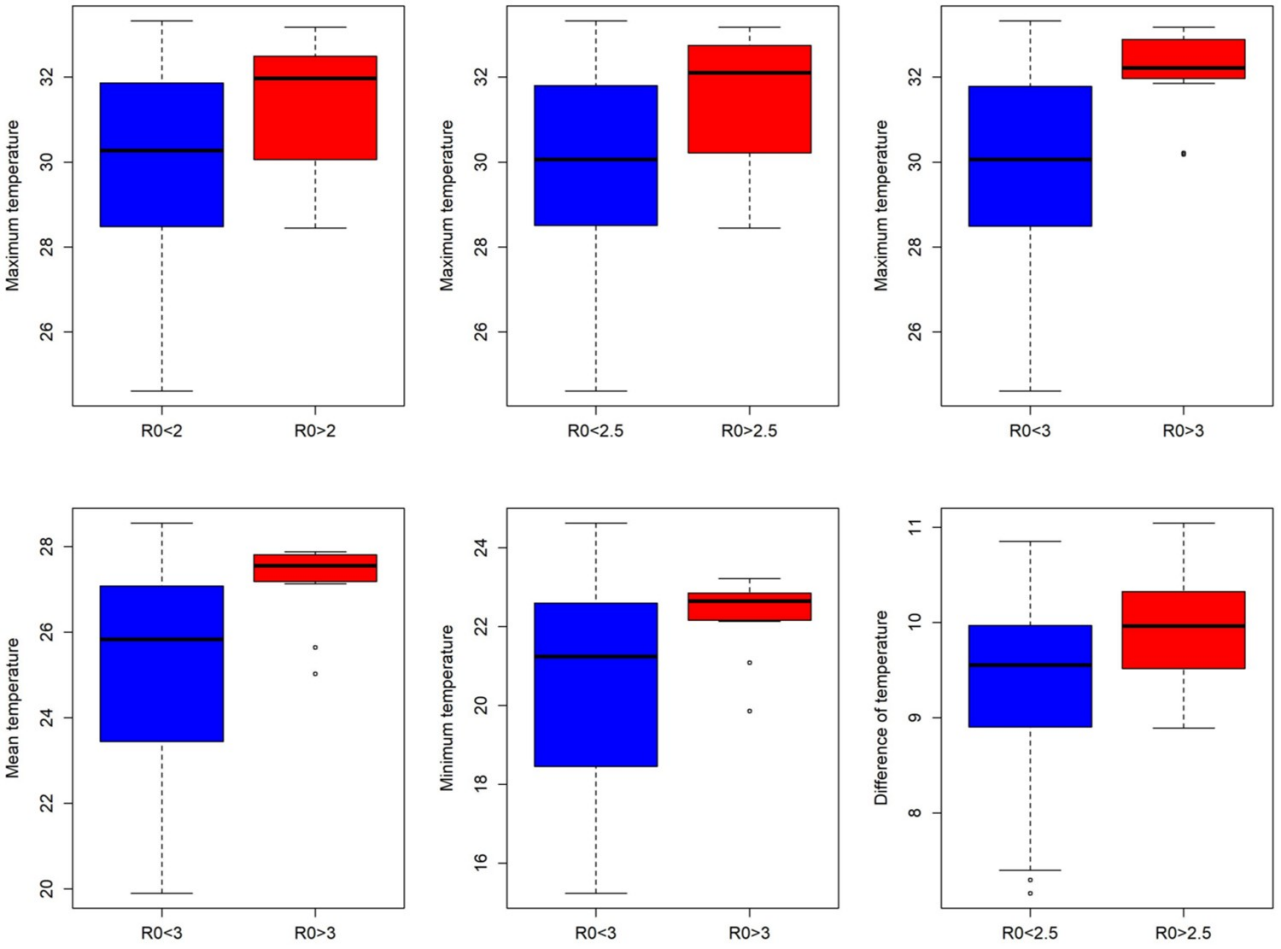
Distribution of values of variables related to temperature of the municipalities with significant differences when they are separated by a *R*_0_ value threshold of 2, 2.5 or 3.

**Fig 4:**
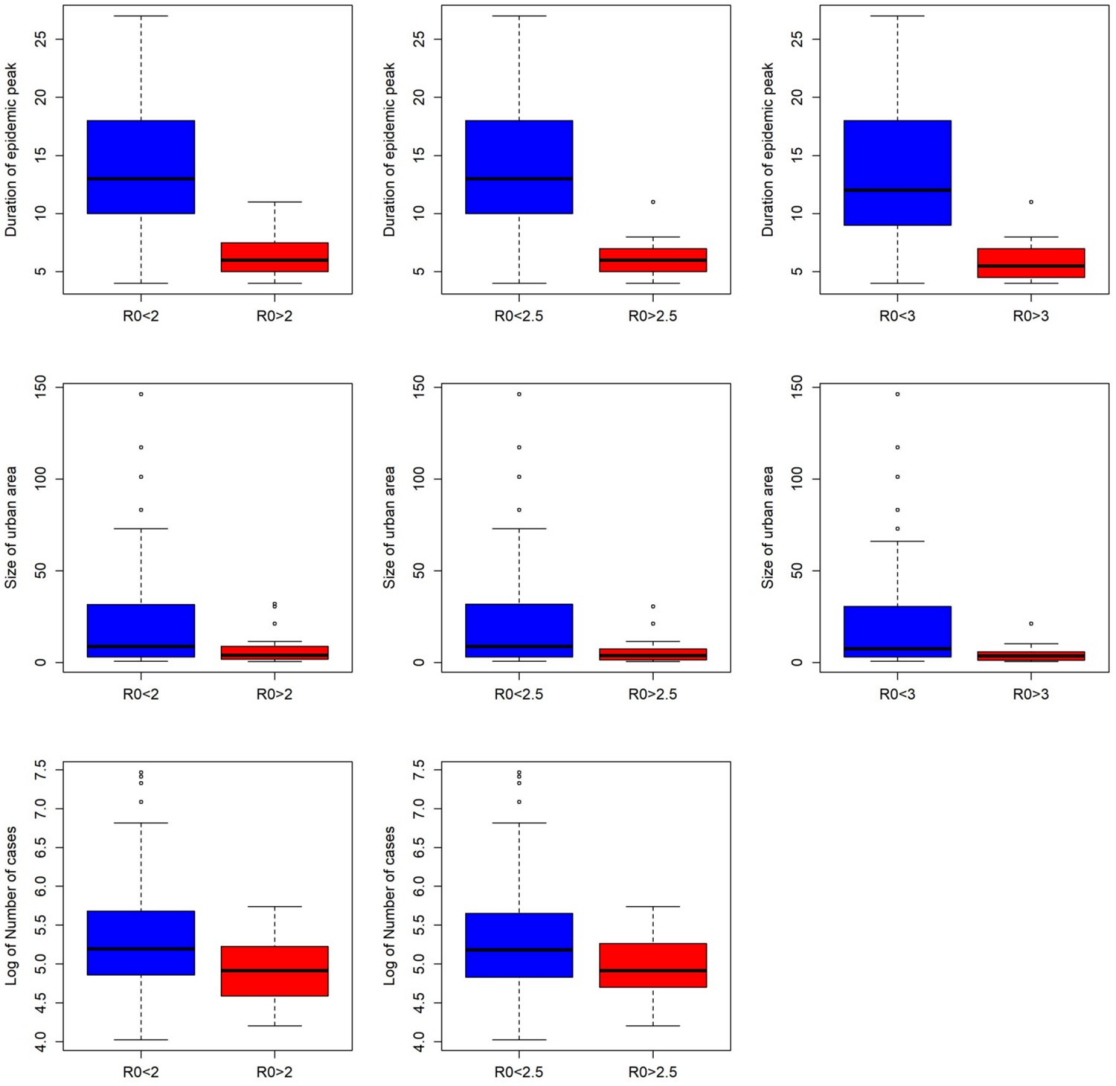
Distribution of values of different variables of municipalities with significant differences when they are separated by a *R*_0_ value threshold of 2, 2.5 or 3.

Population size was not statistically associated with differences in *R*_0_ estimates, and neither was the number of people in the urban center, incidence during the epidemic, population density, or the outbreak onset. Interestingly, both the duration of the epidemic and the size of the urban area were statistically significant in all comparisons (Figs 3 and 4).

Maximum temperature was positively correlated and consistently differential among comparisons. Fig 3 shows this relationship where those municipalities in the lower group had a tendency towards lower temperatures than those municipalities in the higher *R*_0_. Interestingly, the total number of cases and size of the urban area were negatively correlated and consistently statistically different among lower threshold *R*_0_ groupings. Surprisingly, lower *R*_0_ groupings had a tendency to have higher incidence of CHIKV. The size of the urban area was also negatively and consistently different, with higher *R*_0_ groupings having smaller urban areas. Finally, the duration of the epidemic tended to be shorter in those municipalities with higher *R*_0_ estimates (Fig 4).

## Discussion

The White-Pagano methodology allows for estimation of both the serial interval and *R*_0_ using administrative level, epidemiological data [20]. This method is ideal for studies that rely on administrative level data, as it does not require *a priori* estimation of parameters related to mosquito populations, which may be needed on a per-municipality basis [26]. Using this method, we estimated that in municipalities that account for over 64% of the CHIKV cases in Colombia, the *R*_0_ ranged from 1.11 to over 9. This wide range indicates heterogeneity in transmission that, since Colombia was assumed to be a completely susceptible population, could have been related to other factors than the lack of herd immunity.

We investigated several variables that have been associated with arbovirus transmission (and thus *R*_0_) including temperature, urbanicity, and epidemiological characteristics [13, 27–30]. We confirmed what other studies have demonstrated, that higher temperatures (as measured by maximum temperature) is associated with several transmission factors that could lead to higher values of *R*_0_ [12, 18, 27, 31–37]. Though we consider a spectrum of environmental, ecological, and social variables, it is important to note that many other variables likely affect transmission and reporting of cases at different spatial resolutions. Variables such as housing type/quality, income levels, water availability, or access to health services likely all play a role in risk of exposure to arboviruses [38, 39]. However, mosquito density and other socio-ecological variables vary across and within municipalities and are not readily available at the country-level and thus the inclusion of these specific types of variables is of limited value to our model, but urban area size and density of population, included in our study, have been used as proxies for some of these other factors, such as mosquito population [38].

The finding that higher *R*_0_ is associated with smaller urban areas is interesting, especially given that there was a lack of consistently significant relationship between *R*_0_ and the total population of the urban area, the total municipality population, and the density of population indicated. Previous work had revealed that transmission by *Aedes aegypti* is positively associated with intermediate levels of human movement [40]. Particularly with CHIKV, there is evidence in Dhaka, Bangladesh that viral spread is mainly driven by transmission happening at distances that do not exceed neighborhood distances [41]. Our results indicate that higher *R*_0_ values are associated with smaller urban areas, which could be indicative of limited movement of humans in that area. The result is highly focal transmission, which has been reported for CHIKV [42].

**Fig 5:**
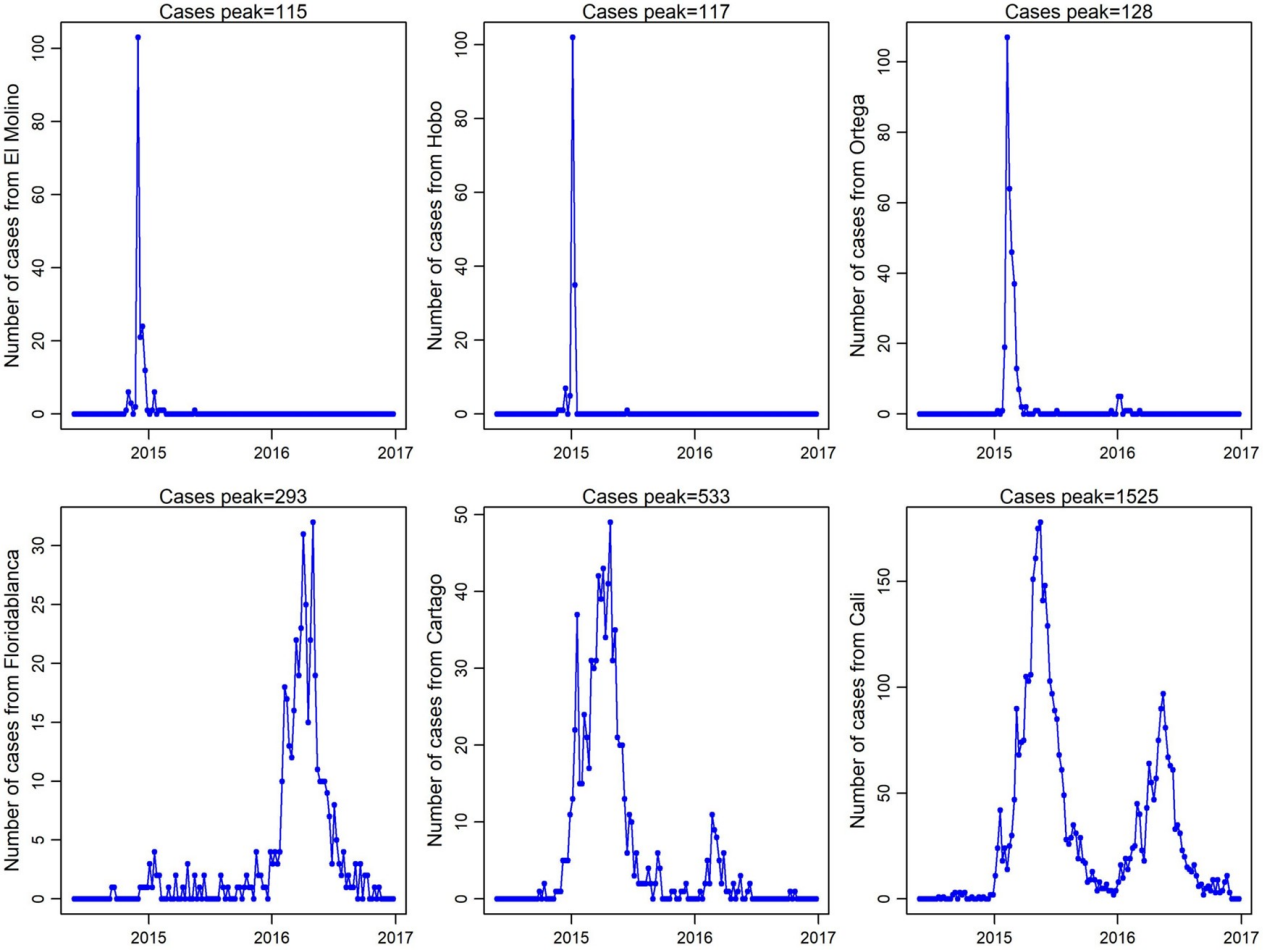
Epidemic peak of three municipalities with the highest (upper) and the three municipalities with lowest (below) *R*_0_ estimated. Above each plot, the cumulative case count from the start until the highest outbreak peak is given.

Of interest was the negative correlation of *R*_0_ grouping with urbanicity, CHIKV case counts, and outbreak time-to-peak. That is, those municipalities with higher values of *R*_0_ showed an explosive outbreak with a fast increase of the number of CHIKV cases but a shorter time-to-peak. These higher *R*_0_ municipalities also had overall lower case counts at the time of peak compared to municipalities that displayed lower values of *R*_0_, where the epidemic continued for many weeks with smaller peak values, but ultimately higher case counts. This phenomenon can be seen in Fig 5, where we plotted the course of epidemic of the three municipalities with highest values of *R*_0_ and the three municipalities with the lowest values of *R*_0_ (but still ≥1).

In summary, this work suggests that CHIKV outbreaks are affected by high maximum temperatures and smaller urban areas, which leads to higher estimates of *R*_0_. These higher *R*_0_ values are then associated with explosive outbreaks with shorter times-to-peak and higher case counts at time of peak (Fig 5). This explosive nature of the initial outbreak leads to a lower overall case count compared to those outbreaks that had longer times-to-peak, lower peak-case counts, but cumulatively larger total number of cases. The tortoise-or-the-hare (TotH) model was proposed to also explain differences in infectious dynamics of arboviruses whereby arboviruses that adopt the tortoise strategy (long-lasting, moderate viremia) will persist longer than arboviruses that adopt the hare strategy (short-lived, high viremia) [43]. We show here that this TotH model, previously used to describe micro-dynamics, may also be suited to describe macro-level dynamics of arboviruses. In theory, the tortoise model would seem to be most advantageous for the virus, as it ultimately achieves the longest and most productive transmission. However, in the context of active surveillance and response where vector control may be activated and result in abrupt cessation or decrease in transmission, the hare model may ultimately represent the most fit population-level phenotype [40, 41]. That is, in areas where control is implemented–such as in Brazil during the Zika epidemic, or Miami during local transmission there–a faster “out-of-the-gate” phenotype (the hare) may result in more transmission, which could be interpreted as the most fit phenotype in this scenario [44, 45]. The TotH model of how to think about transmission represents an interesting relationship between perceived transmission intensity and the ultimate magnitude of transmission (i.e. number of cases). Thus, these findings could impact response prioritization, especially in areas where resources are limited. That is, more intense transmission may appear to be the most pressing concern, when in reality the less intense situations may need more intervention… And given the association of *R*_0_ with eco-environmental variables, the TotH relationship may further have use in predicting allocation of resources at a focal level.” Our findings suggest that the non-monotonic relationship between arbovirus transmission and temperature [46] can be at least in part detected with administrative level case counts and the relationships to the lower bounds of *R*_0_. And given the association of *R*_0_ with eco-environmental variables, the TotH relationship may further have use in predicting allocation of resources at a focal level.

## Supporting information

S1 Fig Frequency of probability values of the 85 municipalities of having one- and two-weeks serial interval. (JPG)

S2 Fig Frequency of CHIKV *R*_0_ values across 85 Colombian municipalities during epidemic peak in 2014–2016. (JPG)
