# Supplementary figures and images for: Correlation of the basic reproduction number (R0) and eco-environmental variables in Colombian municipalities with chikungunya outbreaks during 2014-2016
Source: PLoS Negl Trop Dis. 2019 Nov 7;13(11):e0007878. doi: 10.1371/journal.pntd.0007878 (PMC6863562; doi:10.1371/journal.pntd.0007878)

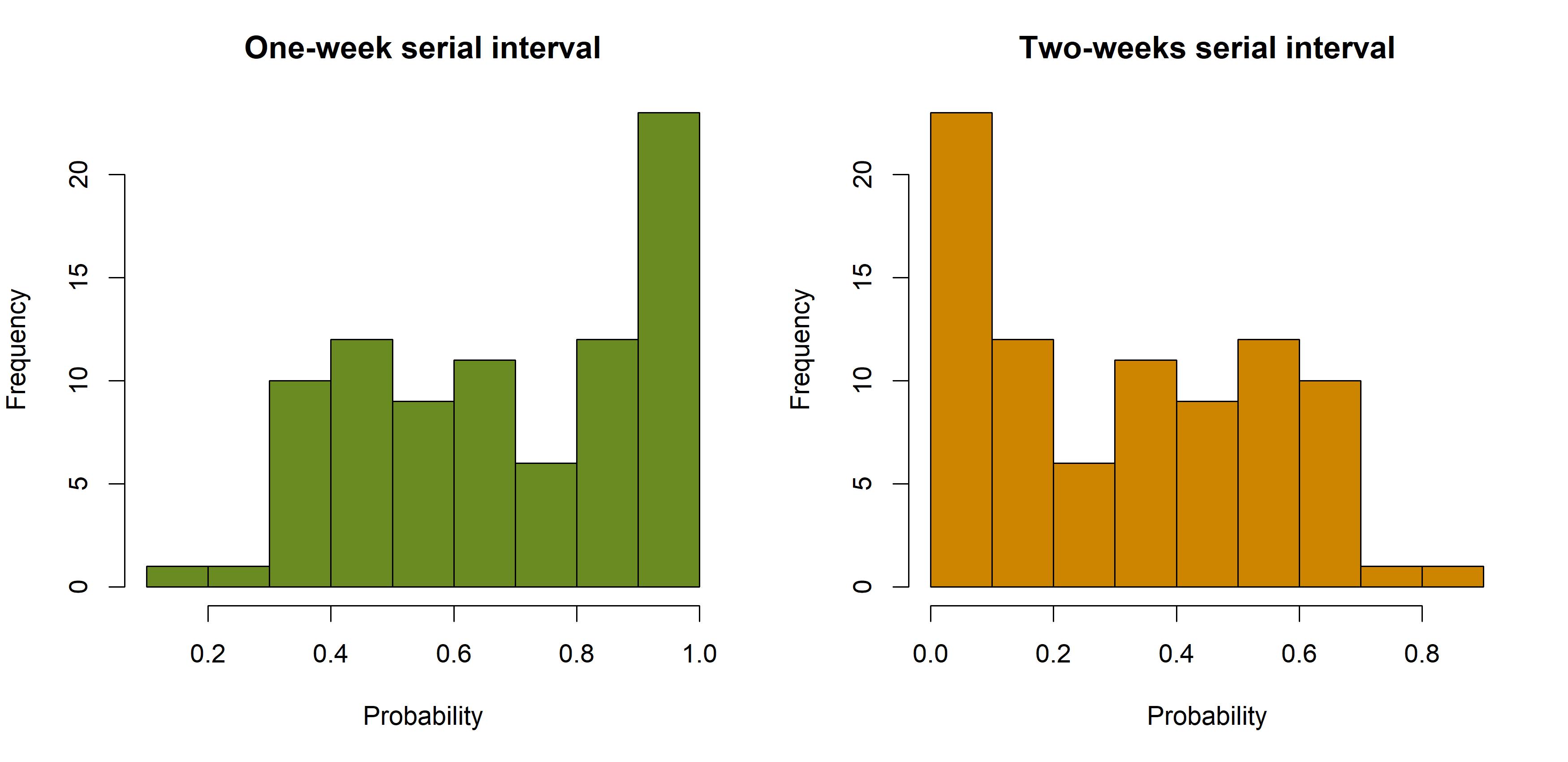

Supplement: S1 Fig — (JPG) [file pntd.0007878.s001.jpg]

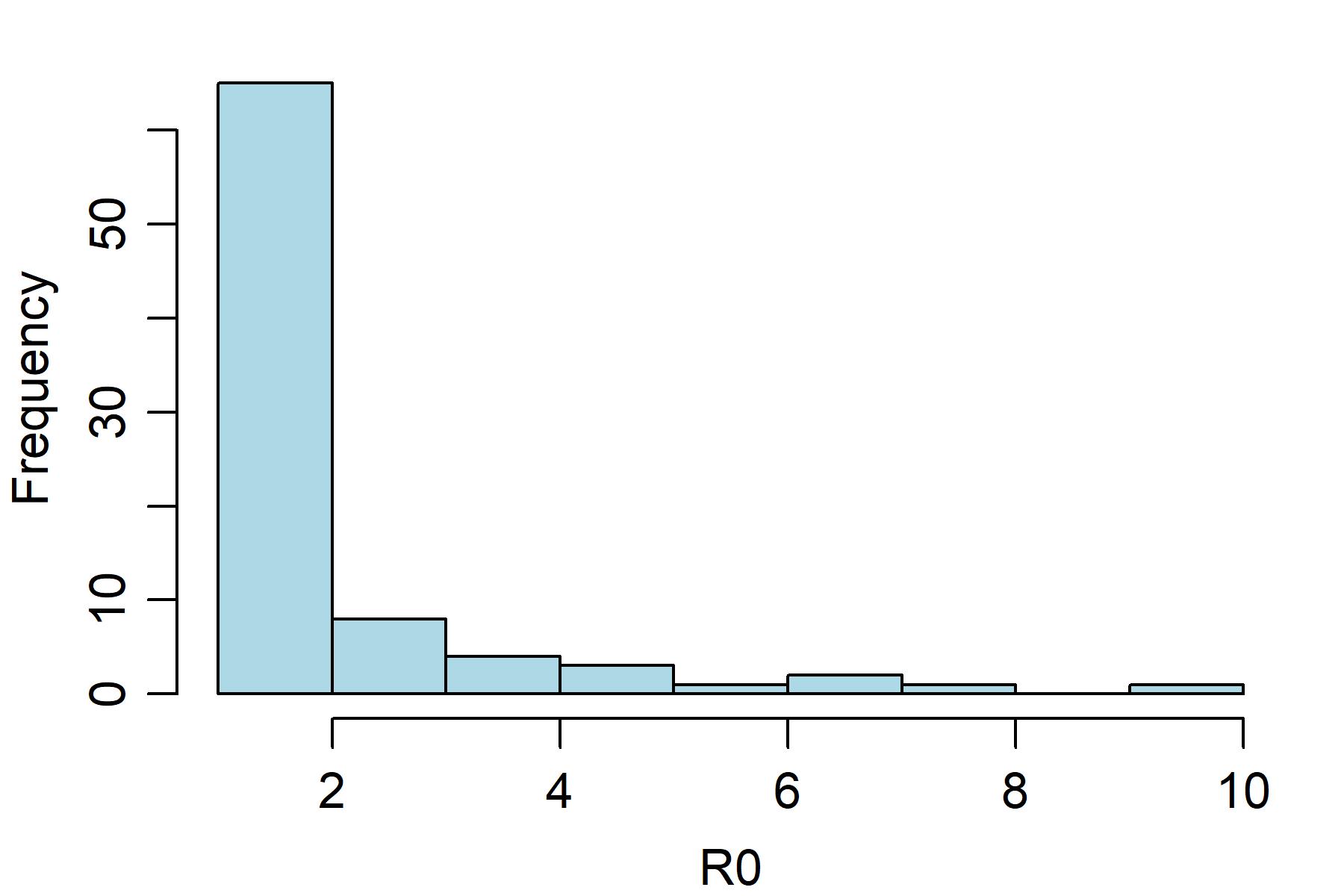

Supplement: S2 Fig — (JPG) [file pntd.0007878.s002.jpg]
